# Normative connectome-based analysis of sensorimotor deficits in acute subcortical stroke

**DOI:** 10.3389/fnins.2024.1400944

**Published:** 2024-08-09

**Authors:** Karolin Weigel, Carsten M. Klingner, Stefan Brodoehl, Franziska Wagner, Matthias Schwab, Daniel Güllmar, Thomas E. Mayer, Felix V. Güttler, Ulf Teichgräber, Christian Gaser

**Affiliations:** ^1^Department of Neurology, Jena University Hospital, Jena, Germany; ^2^Biomagnetic Center, Jena University Hospital, Jena, Germany; ^3^Medical Physics Group, Institute of Diagnostic and Interventional Radiology, Jena University Hospital, Jena, Germany; ^4^Section Neuroradiology, Institute of Diagnostic and Interventional Radiology, Jena University Hospital, Jena, Germany; ^5^Institute of Diagnostic and Interventional Radiology, Jena University Hospital, Jena, Germany; ^6^Department of Psychiatry and Psychotherapy, Jena University Hospital, Jena, Germany; ^7^German Center for Mental Health (DZPG), Department of Psychiatry and Psychotherapy, Jena University Hospital, Jena, Germany

**Keywords:** acute ischemic stroke, brain connectivity, normative connectome, sensorimotor deficits, NIHSS score

## Abstract

The interrelation between acute ischemic stroke, persistent disability, and uncertain prognosis underscores the need for improved methods to predict clinical outcomes. Traditional approaches have largely focused on analysis of clinical metrics, lesion characteristics, and network connectivity, using techniques such as resting-state functional magnetic resonance imaging (rs-fMRI) and diffusion tensor imaging (DTI). However, these methods are not routinely used in acute stroke diagnostics. This study introduces an innovative approach that not only considers the lesion size in relation to the National Institutes of Health Stroke Scale (NIHSS score), but also evaluates the impact of disrupted fibers and their connections to cortical regions by introducing a disconnection value. By identifying fibers traversing the lesion and estimating their number within predefined regions of interest (ROIs) using a normative connectome atlas, our method bypasses the need for individual DTI scans. In our analysis of MRI data (T1 and T2) from 51 patients with acute or subacute subcortical stroke presenting with motor or sensory deficits, we used simple linear regression to assess the explanatory power of lesion size and disconnection value on NIHSS score. Subsequent hierarchical multiple linear regression analysis determined the incremental value of disconnection metrics over lesion size alone in relation to NIHSS score. Our results showed that models incorporating the disconnection value accounted for more variance than those based solely on lesion size (lesion size explained 44% variance, disconnection value 60%). Furthermore, hierarchical regression revealed a significant improvement (*p* < 0.001) in model fit when adding the disconnection value, confirming its critical role in stroke assessment. Our approach, which integrates a normative connectome to quantify disconnections to cortical regions, provides a significant improvement in assessing the current state of stroke impact compared to traditional measures that focus on lesion size. This is achieved by taking into account the lesion’s location and the connectivity of the affected white matter tracts, providing a more comprehensive assessment of stroke severity as reflected in the NIHSS score. Future research should extend the validation of this approach to larger and more diverse populations, with a focus on refining its applicability to clinical assessment and long-term outcome prediction.

## 1 Introduction

Stroke, as the second leading cause of death and the leading cause of acquired disability, remains a major burden in Western countries (Katan and Luft, 2018; Gbd 2019 Stroke Collaborators, 2021). Over the past decade, substantial advancements have been made in both diagnostic and therapeutic strategies for acute stroke. Perfusion imaging has emerged as a vital tool, offering insights into potentially salvageable at-risk tissue. Acute treatment modalities, notably thrombolysis therapy and mechanical thrombectomy, hold tremendous promise. However, they are not without risks, necessitating a careful evaluation of the potential benefits against inherent risks.

One of the challenges in acute stroke management is the absence of a reliable method to assess the future clinical implications of a specific lesion. The inability to accurately predict untreated outcomes, both in the immediate aftermath and over the long term, complicates acute treatment decisions. These studies are mostly based on either the National Institutes of Health Stroke Scale (NIHSS score) or the volume of brain tissue at risk (Goyal et al., 2016; Ma et al., 2019). However, more detailed knowledge of the potential harm of a particular lesion would be extremely useful.

The critical need to address this issue has spurred a plethora of studies aiming to predict patients’ clinical outcomes, employing various data types. These approaches can be broadly categorized into three main strategies: (i) analysis of pure clinical data, (ii) assessment of lesion size and location, and (iii) integration of network connectivity information.

Efforts to forecast clinical outcomes based solely on clinical data have yielded moderate results, indicating that patients with more severe initial symptoms generally experience poorer outcomes (Sato et al., 2008; Sablot et al., 2011; Wouters et al., 2018; Kazi et al., 2021). Another approach involves incorporating the size and location of the lesion in outcome prediction models. Initially, this approach was expected to enhance prediction accuracy significantly, as the lesion directly contributes to clinical symptoms and potential persistent disability. However, despite considerable efforts, the results have not reached a level of accuracy applicable in clinical practice (Schiemanck et al., 2005; Yoo et al., 2010). This limitation extends to other parameters in prediction models, such as cortical thickness in certain contralesional cortices (Rojas Albert et al., 2022) and metrics of brain age and resilience (Liew et al., 2023).

To obtain data on functional or structural connectivity, resting state functional magnetic resonance imaging (rs-fMRI), diffusion tensor imaging (DTI) and connectivity analyses have been employed (Horn et al., 2014; Ktena et al., 2019; Kao et al., 2020; Koch et al., 2021; Peng et al., 2023). However, these data (rs-fMRI, DTI) are not typically gathered during routine diagnostics in stroke cases. Moreover, obtaining them requires patient cooperation and extends the examination time, rendering them impractical in the hyperacute phase of stroke, where therapeutic decisions are time-sensitive. In acute stroke diagnostics, a diffusion sequence and FLAIR are necessarily acquired in MRI. Our approach utilizes a T1 and FLAIR data set, whereby even the T1 could be generated from the FLAIR (Iglesias et al., 2023). This emphasis on basic MRI sequences reduces the MRI acquisition time to just several minutes. Consequently, systemic thrombolysis and mechanical thrombectomy can be rapidly initiated during the hyperacute phase of stroke.

Our alternative approach combines individual MRI data with probabilistic information sourced from the Human Connectome Project (HCP). We hypothesized that the clinical state of a patient can be predicted using probabilistic information from the HCP, rather than individual DTI data. Specifically, we posit that this connectome information could enhance the predictability of clinical stroke severity, as measured by the NIHSS score, beyond what can be achieved by considering lesion size alone. To estimate the number of fibers traversing the lesion, we calculated the number of disconnected fibers and used the term disconnection value. In order to evaluate the hypothesis, we assessed the number of disconnected fiber tracts to specific cortical areas (quantified by the disconnection value), drawing on probabilistic data from the HCP alongside individual T1 and T2-weighted fluid-attenuated inversion recovery (FLAIR) imaging data from our cohort of 51 patients.

## 2 Materials and methods

The study was reviewed and approved by the Ethics Committee of Friedrich Schiller University Jena in accordance with the Declaration of Helsinki.

We employed a uniform and standardized examination design for all patients included in the project. Stroke patients underwent clinical examinations, and their medical histories were reviewed. This procedure, as well as asking specific questions within the framework of the anamnesis, is well-established. In addition, we developed a software that utilizes data provided by the HCP along with a high-resolution image of the patient’s ischemic lesion to create a probabilistic surface atlas of the disconnected areas.

### 2.1 Patients

Fifty-one stroke patients (aged 47 to 88 years, mean age 69.0 ± 10.5; 24 female) were screened at the stroke unit and intensive care unit within the Department of Neurology of the University Hospital Jena, Germany. We analyzed data from a two-year period, starting in December 2019 and ending in December 2021. After presentation to the Emergency Department the patients were admitted to our certified stroke unit or neurologically managed intensive care unit. They received an interdisciplinary stroke unit treatment over at least 24 h.

The following inclusion criteria were defined: (a) acute or subacute single subcortical ischemic stroke in anterior, media, or posterior territory visible in cranial magnetic resonance imaging (MRI) not older than 5 days, (b) focal neurological symptoms (motoric and/or sensoric) caused by the stroke, (c) NIHSS score under 15 points at the time of admission, (d) aged 18 years or older, and (e) no evidence of other neurological disorders to explain the symptoms.

In addition to sociodemographic data, we documented a range of parameters and clinical information, including the CHA2DS2-VASc score, which assigns 1 point for congestive heart failure, hypertension, age 65–74 years, diabetes mellitus, vascular disease, female sex and 2 points for age > 75 years and previous stroke, transient ischemic attack or thromboembolism (January et al., 2014). Long-term medication, such as oral anticoagulation, and (dual) platelet aggregation inhibition, were also carefully recorded. Acute therapeutic interventions, such as systemic thrombolysis, mechanical thrombectomy, and acute carotid artery thromboendarterectomy, were documented, along with the duration of hospital stay (comprehensive details available in Table 1).

**TABLE 1:** Cohort characteristics.

N	51
Age in years	69.0 ± 10.5 (47–88)
Females (in %)	24 (47.1%)
NIHSS score admission	4.4 ± 3.2 (0–14)
NIHSS score release (early follow-up)	2.1 ± 2.6 (0–10, N = 50)
mRS (pre-stroke level)	0.5 ± 0.9 (0–3, N = 48)
mRS release (early follow-up)	1.8 ± 1.2 (0–4, N = 47)
Oral anticoagulation in premedication (in %)	2 (3.9%)
Platelet aggregation inhibition in premedication (single or dual; in %)	14 (27.5%)
Affected hemisphere (right vs. left in %)	20 vs. 31 (39.2% vs. 60.8%)
CHA2DS2-VASc score	4.8 ± 1.6 (2–8)
Systemic thrombolysis (in %)	10 (19.6%)
Mechanical thrombectomy (in %)	4 (7.8%)
Acute carotid artery thrombendarterectomy (in %)	0 (0%)
Duration of hospital stay in days	7.0 ± 3.0 (2–15)

NIHSS, National Institutes of Health Stroke Scale; mRSm modified Rankin Scale, CHA2DS2-VASc score [acronym stands for congestive heart failure, hypertension, age in years (>65 = 1 point, >75 = 2 points), diabetes mellitus, vascular disease, female sex and previous stroke/transient ischemic attack/thromboembolism (2 points)]. Values are given in mean value ± standard deviation (range).

We focused on relatively less severely affected stroke patients to make participation in the study practically possible. By definition, these were patients with mild or moderate stroke (determined by NIHSS score < 15 points). Specifically, this was determined by the NIHSS score ranging from 0 to 6 points for mild stroke and 7–14 points for moderate stroke. Patients with severe clinical deficits often had complicated courses, which made inclusion in the study impossible (e.g., due to prolonged stays in the intensive care unit).

Patients meeting any of the following criteria were excluded from the study: the presence of acute visual defects (such as hemianopsia), pre-infarcts in the same cerebral area affected by the current stroke, pre-existing motor or sensory deficits related to stroke, and any contraindications to MRI, e.g., patients having a pacemaker.

### 2.2 Clinical assessment

Neurological deficits were quantified using the NIHSS score, which is suitable for early detection and follow-up assessment (Brott et al., 1989; Kasner, 2006). The NIHSS score was recorded by trained neurologists (assistant and specialist doctors) at the time of admission. To determine the degree of disability after stroke, the modified Rankin Scale (mRS) score was utilized in the currently widely modification (Rankin, 1957; van Swieten et al., 1988).

### 2.3 Magnetic Resonance Imaging (MRI)

All patients included in the project underwent MRI scanning, which included acquiring a high-resolution T1 dataset with an isotropic voxel size of 1 mm and a FLAIR sequence with identical resolution. The MRI scans were conducted using a 3 Tesla MRI scanner (Skyra, SIEMENS, Erlangen, Germany) approximately 2–5 days post-hospitalization (average 2.6 days). A standardized MRI protocol was employed for all scans, encompassing both a T1-weighted and FLAIR sequence. The high-resolution T1-weighted anatomical dataset was acquired using a three-dimensional (3D) magnetization-prepared, rapid acquisition gradient-echo (MPRAGE) sequence with the following parameters: voxel size of 1 mm isotropic, acquiring 176 sagittal slices with a slice thickness of 1 mm. The repetition time (TR) was 2300 milliseconds (ms), echo time (TE) 3.06 ms and inversion time (TI) 1,100 ms. Additionally, FLAIR images were captured with the following parameters: voxel size of 1 mm isotropic, 176 sagittal slices, slice thickness 1 mm. TR = 5000 ms, TE = 394 ms, TI = 1,800 ms.

### 2.4 Image processing and data evaluation

**FIGURE 1:**
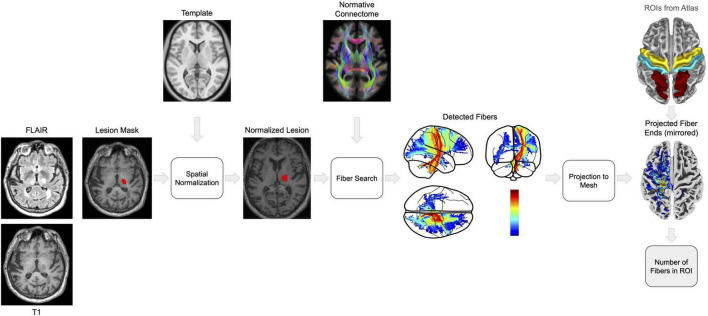
The figure shows a step-by-step overview for processing MRI scans and mapping fiber tracts to investigate the effects of stroke lesions on brain connectivity. We start with co-registration of FLAIR to T1-weighted images using SPM12 and utilize the derived parameters to align lesion masks. Subsequently, the Lesion Segmentation Toolbox (LST) is employed for lesion identification and filling on T1 images, a process designed to mitigate the bias of spatial normalization to the MNI152NLin2009cAsym space with CAT12. The HCP1065 Population-Averaged Tractography Atlas then facilitates the identification of fiber tracts intersecting the lesions. These fibers are projected onto a cortical mesh in MNI space. To ensure consistent analysis across subjects, lesion projections from the right hemisphere are mirrored to the left hemisphere. Affected cortical regions, specifically the precentral, postcentral, and superiorparietal areas deliniated by the Desikan-Killiany (DK40) atlas, are quantified by the logarithmically (log) scaled number of terminating fibers, elucidating the extent of disconnection following stroke. To obtain the disconnection value, we multiplied the total number from the three specified regions. The color spectrum in the fiber detection and projection images serves as an indicator of disconnection: warmer tones denote a greater number of severed fibers, whereas cooler tones indicate fewer disconnections.

The raw datasets contained the T1 and FLAIR images. Ischemic lesions were detected in the FLAIR sequence. All images were pre-processed using Statistical Parametric Mapping (SPM12, Friston et al., 2007) and the Computational Anatomy Toolbox (CAT12, Gaser et al., 2022). The following Figure 1 gives an overview of the schematic workflow:

#### 2.4.1 Preprocessing of images

To create lesion masks from the stroke patients, we used in-house software under the guidance of expert supervision. The following steps were performed:

(1) Labeling: We assigned the existing image data to the respective correct image modality.
(2) Preprocessing: We changed the orientation (rotation from sagittal to axial) and marked the commisura anterior for correct normalization. If the MRI images appeared misaligned, additional alignment along an angle was executed according to radians (especially important for very oblique images). Manually, we set the course of the axis along the falx cerebri.
(3) Detection and marking regions of interest (ROIs): Every image underwent thorough visual inspection. Acute ischemic lesions were located and marked by delineating the entire lesion on each layer, utilizing the FLAIR dataset as reference.

After creating the lesion masks, we co-registered the FLAIR images to the T1-weighted image using normalized mutual information (NMI) in SPM12. The estimated registration parameters were then applied to the lesion masks. Following this, we used the Lesion Segmentation Tool (LST, Schmidt and Wink, 2017) employing the lesion growth algorithm (LGA, Schmidt et al., 2012) approach to accurately segment the stroke lesions, utilizing the default parameters. Subsequently, the segmented lesions were filled in the T1 image using the LST lesion filling function. Our preference for LST over manually defined lesions stems from its ability to not only fill stroke lesions but also address other lesions in the white matter. This ensured the creation of a lesion-filled T1 image, mitigating any potential biases induced by lesions in estimating spatial registration. This lesion-filled T1 image served as the basis for estimating the deformations from the T1 image to the standardized template space MNI152NLin2009cAsym using CAT12. Finally, the deformations were applied to the co-registered stroke lesion mask, facilitating the transformation of all images into MNI152NLin2009cAsym space.

#### 2.4.2 Fiber projection using normative connectome

**FIGURE 2:**
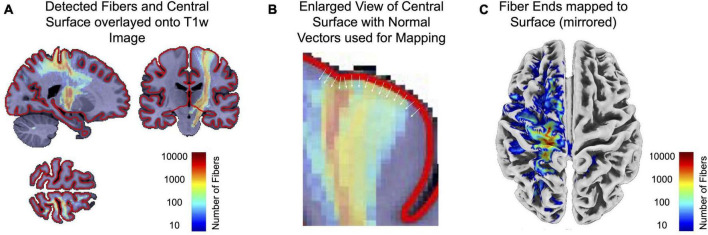
This figure illustrates a more detailed description of the use of the normative connectome atlas. The used HCP1065 atlas provides coordinates for 0.5 million fiber tracts, resampled to match our 1.5 mm voxel size. Each voxel now contains a count of fibers, log-transformed for standardization. This helps to identify fibers that cross lesions. Fibers traversing lesions are identified, creating a map **(A)** showing their full length and endpoints. The CAT12 surface mesh of the MNI152NLin2009cAsym template now maps voxel values using inward surface normals to find maximum values within a given depth **(B)**. The resulting surface map **(C)** finally encodes the number of fibers traversing lesions. Again, values are stored as log-transformed values to account for the wide range of values.

To identify fiber tracts traversing the stroke lesions, we utilized the HCP1065 Population-Averaged Tractography Atlas (Yeh, 2022). This probabilistic tractography atlas, constructed from 1065 DTI scans, provides a normative map of fiber tracts transformed into MNI152NLin2009cAsym space. It comprises 0.5 million fiber tracts, manually corrected and curated for quality assurance. The atlas is publicly accessible.^1^ As the lesions now align with the HCP1065 atlas, we could determine which fibers traversed each lesion in each subject and calculate a disconnection value for each patient using the following steps:

(1) The original HCP1065 atlas records precise coordinates in MNI152NLin2009cAsym for each of the 0.5 million fiber tracts. These coordinates are resampled to match the 1.5mm voxel size of our spatially registered (lesion) images. Each voxel now contains the count of fibers passing through it. To standardize the wide range of these values and reduce distribution skewness, we applied a logarithmic (log) transformation. For instance, a value of 3 indicates that 1000 fibers traverse the voxel. Notably, all voxel coordinates for all fiber tracts are retained after this resampling, facilitating the identification of fibers traversing a lesion in subsequent steps.
(2) All fibers intersecting the lesion are identified, with each voxel containing information on the number of fibers crossing it. This produces a map (Figure 2A) illustrating the full length of fibers traversing the lesion, including their endpoints.

(3) CAT12 provides a surface mesh of the MNI152NLin2009cAsym template, utilized to map voxel values from the previous step, containing information on the number of fibers present in each voxel. Surface normals pointing inward are employed to find the maximum value within a specified depth (Figure 2B). This mapping process is analogous to the mapping of functional MRI data to the surface, but it extends the search depth to encompass values close to the gray-white matter boundary.
(4) The resulting surface map (Figure 2C) now encodes the number of fibers found in the underlying voxels, representing fibers traversing the lesion and terminating at specific surface points. Once again, values are stored as log-transformed values to account for the wide range of values.

**FIGURE 3:**
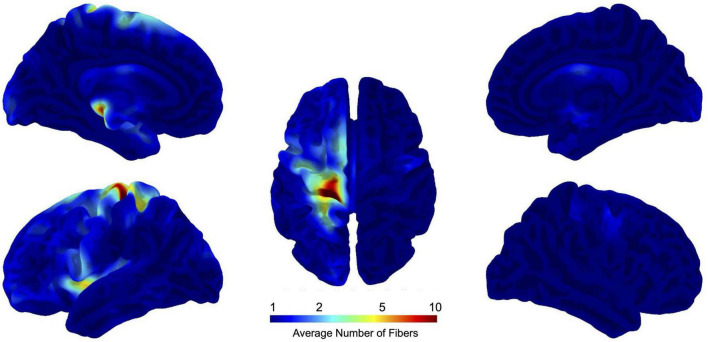
This figure illustrates the mapping of the average number of fibers intersecting stroke lesions in 51 stroke patients. The data are projected onto a standardized brain template in MNI152NLin2009cAsym space, allowing precise localization of affected brain regions. Logarithmic scaling is used to manage the wide range of fiber counts, allowing more interpretable visualization. A value on this scale means that, on average, 10 fibers overlap a given point on the brain surface. The projection highlights the cortical areas most affected by the stroke, providing insight into the impact of the lesion on brain structure. This technique makes it possible to quantify the extent of disconnection in specific regions.

In order to facilitate the analysis of all stroke lesions in the same hemisphere, lesions located in the right hemisphere were mirrored to the symmetrical left hemisphere (see Figures 1, 2). The average log-scaled fiber numbers across our sample of 51 patients are shown in Figure 3. These projected fiber endpoints serve as the basis for calculating the logarithmic number of fibers terminating in each of the precentral, postcentral, and superior parietal regions of the Desikan-Killiany (DK40) atlas (Desikan et al., 2006), thereby providing insight into the affected cortical areas. The total number of detected fibers is estimated for each of these regions.

For subsequent statistical analysis, we multiplied rather than summed the log-scaled fiber counts from the three specified regions, which now represents our disconnection value. This approach ensures that all three regions are affected simultaneously, emphasizing the pervasive effect of stroke on the targeted cortical regions. By using this multiplicative approach, we ensure that the resulting values are significant only when fibers are present in each of the three ROIs, thereby highlighting regions that are profoundly affected by stroke across all cortical regions targeted in this study.

To summarize the entire calculation of this disconnection value in one equation:

Disconnection⁢value=

$$\prod_{r\in \left\{s\,u\,p\,e\,r\,i\,o\,r\,p\,a\,r\,i\,e\,t\,a\,l,p\,o\,s\,t\,c\,e\,n\,t\,r\,a\,l,p\,r\,e\,c\,e\,n\,t\,r\,a\,l\right\}}\left(\sum_{i\in L\,e\,s\,i\,o\,n\,\left(r\right)}l\,o\,g\,\left(n_{f\,i\,b\,e\,r\,s,i}+1\right)\right)$$

where *n*_*fibers,i*_ represents the number of fibers at position *i*, $\sum_{i\in L\,e\,s\,i\,o\,n\,\left(r\right)}l\,o\,g\,\left(n_{f\,i\,b\,e\,r\,s,i}+1\right)$ is the sum of the log-scaled fiber counts (incremented by one to ensure that logarithm is defined) within each specified region r, and $\prod_{r\in \left\{s\,u\,p\,e\,r\,i\,o\,r\,p\,a\,r\,i\,e\,t\,a\,l,p\,o\,s\,t\,c\,e\,n\,t\,r\,a\,l,p\,r\,e\,c\,e\,n\,t\,r\,a\,l\right\}}$ describes the product of the sums of log-scaled fiber counts across three regions, reflecting the combined effect of disconnection in multiple areas.

#### 2.5 Statistical testing

To evaluate the importance of the predictor variables, lesion size and disconnection value, we initially conducted simple linear regression analysis with lesion size as the sole predictor. This initial model served as the baseline for further analyses. Subsequently, we performed hierarchical linear regression analysis to investigate the incremental value of disconnection value in predicting the outcome variable, beyond what can be explained by lesion size alone. Hierarchical linear regression allows for the sequential entry of variables into the regression equation, which is particularly useful for testing the unique contribution of variables in the presence of other variables. The hierarchical linear regression analysis was conducted in two steps: Model 1 (Baseline Model): Included only lesion size as the predictor. Model 2 (Full Model): Included both lesion size and disconnection value as predictors. Analysis of variance (ANOVA) was employed to compare the explanatory power of these models. This analysis tests whether the variance explained by the full model is significantly greater than that explained by the baseline model, effectively evaluating the contribution of adding disconnection value to the prediction of the dependent variable. This method allows us to assess whether the addition of disconnection value significantly improves the model fit and predictive accuracy compared to a model relying solely on lesion size. A significant ANOVA result would suggest that disconnection value provides valuable predictive information beyond lesion size. Further methodological details and examples of hierarchical linear regression in similar contexts can be found in previous work on applied multiple regression/correlation analysis in the behavioral sciences (Cohen et al., 2022) and in the discussion regarding the integration of new predictors into existing regression models (Hayes, 2018).

## 3 Results

In our research study we included 20 right and 31 left hemispheric strokes (*n* = 51; 39.2% vs. 60.8%; 24 females). All patients reported acute focal neurological symptoms before first presentation in the Emergency Department. The mean admission NIHSS score was 4.4 ± 3.2 points (range: 0–14), and the average length of hospital stay was 7.0 ± 3.0 days (range: 2–15).

**FIGURE 4:**
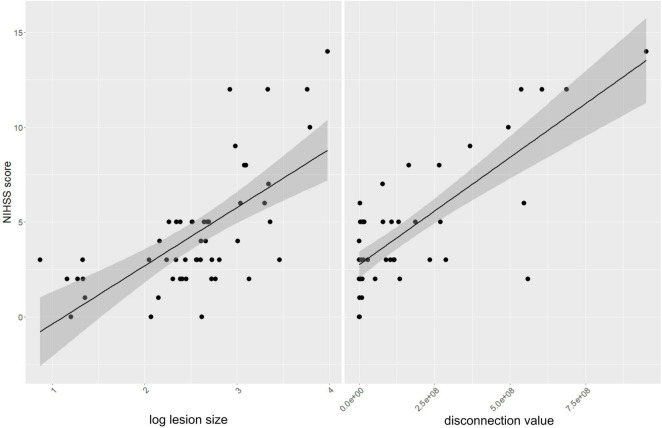
Correlation analysis: The left panel illustrates a linear regression analysis depicting the relationship between the NIHSS scores and the logarithmically transformed lesion sizes. The right panel shows the correlation between NIHSS scores and disconnection values.

To assess the respective importance of lesion size and disconnection value, we initially performed two separate simple linear regression analyses to test if (1) the lesion size and (2) the disconnection value significantly predict the NIHSS score. The model utilizing the lesion size revealed a significant result, explaining 44% of the measured NIHSS score (*R*^2^ = 0.44; adjusted *R*^2^ = 0.43, *F*-value = 38.0; *p* < 0.001). We observed that lesion size significantly predicted the NIHSS score (β = 0.66, *p* < 0.001, see Figure 4 and Table 2 for details). Similarly, the model utilizing the disconnection value also revealed a significant result, explaining 60% of the measured NIHSS score (*R*^2^ = 0.60; adjusted *R*^2^ = 0.59, *F*-value = 72.9; *p* < 0.001). It was found that the disconnection value significantly predicted the NIHSS score (β = 0.77, *p* < 0.001, see Figure 4 and Table 3 for details).

**TABLE 2:** Model 1, simple linear regression of NIHSS score by lesion size (B: unstandardized beta, β: standardized beta, SE B: standard error for unstandardized beta, *p*: probability value).

Predictors	B	SE B	β	*P*
Constant	−3.47	1.32		0.01
Log lesion size	3.08	0.50	0.66	1.3e–7

*R*^2^ = 0.44; Adjusted *R*^2^ = 0.43, *F*-value = 38.0; *P* = 1.3e–7.

**TABLE 3:** Model 2, simple linear regression of NIHSS score by disconnection value.

Predictors	B	SE B	β	*P*
Constant	2.75	0.35		2.38e–10
Disconnection value	1.1e–8	1.3e–9	0.77	2.9e–11

*R*^2^ = 0.60; Adjusted *R*^2^ = 0.59, *F*-value = 72.9; *P* = 2.9e–11.

To further investigate the value of the disconnection value compared to lesion size, we compared the model that uses log lesion size as the sole predictor with a model that includes both log lesion size and the disconnection value as predictors (Model 3, Table 4). The overall regression in Model 3 was statistically significant (*R*^2^ = 0.64; adjusted *R*^2^ = 0.63, *F*-value = 43.1; *p* < 0.001). We found that both predictor variables made a significant contribution to the model (lesion size: β = 0.28, *p* = 0.019; disconnection value: β = 0.60, *p* < 0.001, Table 4). We then compared both models using ANOVA to test whether there is a significant difference between both models in predicting the NIHSS score. Results demonstrated that the addition of the disconnection value significantly improved the fit of the model (*F* = 27.6, *p* < 0.001, Table 5). In other words, the prediction of the NIHSS score could be enhanced significantly by incorporating the disconnection value into the regression model (*p* < 0.001).

**TABLE 4:** Model 3, multiple linear regression of NIHSS score by lesion size and disconnection value.

Predictors	B	SE B	β	*P*
Constant	−0.15	0.12		0.90
Log lesion size	2.29	9.4e–9	0.28	0.019
Disconnection value	8.7–e9	1.7e–9	0.60	3.4e–6

*R*^2^ = 0.64; Adjusted *R*^2^ = 0.63, *F*-value = 43.1; *P* = 1.9e–11.

**TABLE 5:** ANOVA comparing the two linear regression models predicting NIHSS admission scores by only log lesion size (Model 1, Table 2) or by log lesion size and the disconnection value (Model 3, Table 4). The results indicate that adding the disconnection value as a predictor in Model 3 significantly improves the model’s fit compared to Model 1, which only includes the log lesion size (Res.Df: Residual Degrees of Freedom, RSS: Residual Sum of Squares).

Model	Res.Df	RSS	F	*P*
1	49	294.1	–	–
3	48	187.7	27.6	3.4e–6

## 4 Discussion

This study underscores the potential of probabilistic connectome data in predicting the clinical status of stroke patients, showcasing its superiority over methods relying solely on lesion size. Previous research has already established that connectivity-based models, which integrate additional insights about the brain’s functional and structural wiring, offer enhanced predictive power compared to models limited to clinical data or lesion metrics (Grefkes and Fink, 2014; Silasi and Murphy, 2014; Dulyan et al., 2022; Rivier et al., 2023). This advantage aligns with the prevailing network-centric perspective of brain function, recognizing that brain functionalities are not isolated but emerge from complex, widely distributed networks that necessitate constant informational exchange (Siegel et al., 2022). Our findings also resonate with this perspective, illustrating how focal brain lesions can profoundly impact local and global network functions by disrupting not only the directly affected area but also its connected regions (Foulon et al., 2018; Saenger et al., 2018; Griffis et al., 2019; Salvalaggio et al., 2020). This is exemplified by studies on subcortical stroke lesions that indicate their impact extends beyond the immediate site, influencing connected cortical areas and leading to cortical thinning due to secondary neuroaxonal degeneration (Cheng et al., 2015; Duering et al., 2015).

While patient-specific data derived from high-resolution individual DTI and rs-fMRI examinations would offer the most accurate insight into structural and functional connectivity, the practical constraints of routine clinical practice often render this unfeasible. However, our approach, leveraging a probabilistic connectome, necessitates only the lesion’s size and location and yet outperforms methods lacking connectome insights. This implies that probabilistic connectome information provided by an atlas offers valuable features for predicting a patient’s clinical condition. To optimize prediction models, integrating these features into machine learning approaches is crucial, as the quality of input features heavily influences model performance (Rehme et al., 2015; Mouridsen et al., 2020; Jayakumar et al., 2022). Utilizing preprocessed connectome features is essential, as machine learning models cannot infer the probabilistic connectivity structure on a population basis from limited data. It is crucial to emphasize that for a system to be viable for clinical application, it must be fundamentally grounded in data that can be routinely obtained in clinical environments. While advanced methodologies like DTI and functional connectivity analyses greatly enhance our scientific understanding, their applicability in clinical settings may be limited. This limitation becomes particularly pronounced during the critical, high-acuity phase of stroke management, where swift and decisive actions regarding recanalization interventions are paramount. The strength of the approach presented in this study lies precisely in its clinical applicability, leveraging data that are readily available and can be efficiently utilized during the time-sensitive decision-making process inherent in stroke treatment. This ensures that the methodology is not just theoretically robust but also practically implementable, aligning with the real-world constraints and urgent needs of acute stroke care. A primary objective for the future is to facilitate the availability and routine individual application of this innovative approach in everyday clinical practice for stroke patients.

We showed that this method, which uses a normative connectome in predefined cortical ROIs, can significantly improve the prediction of stroke outcome compared to traditional measures of lesion size. This finding is consistent with a growing body of evidence suggesting that damage to white matter tracts plays a more important role in stroke outcome than lesion size alone (Moulton et al., 2019; Kessner et al., 2021). Further, there has been previous work on assessing stroke severity based on lesion size and location. One widely utilized approach is voxel-based lesion-symptom mapping (VLSM), which has generally been used for more specific scores such as aphasia scores (Bates et al., 2003; Park et al., 2021), but can also be applied to the NIHSS score (McCullough-Hicks et al., 2020). Other approaches have focussed on different measures, such as corticospinal lesion load, which is attributed with greater explanatory power than lesion size. A comparative study demonstrated that the extent of functional motor deficits following stroke strongly depends on the degree of lesion overlap with the corticospinal tract rather than lesion size itself (Zhu et al., 2010). Further studies confirmed the predictive value of the lesion load of the corticospinal tract for motor outcomes after stroke (Feng et al., 2015; Ito et al., 2022; Park and Ohn, 2022). In recent times, the John Hopkins University (JHU) white-matter atlas, which comprises volumetric white matter tracts, has found application. Using it as a kind of normative connectome, damaged white matter pathways were identified that could cause a specific deficit (unilateral spatial neglect) after subcortical stroke (Cha et al., 2022). Similarly, the JHU atlas could also be applied to neuronal substrates associated with subcortical aphasia in stroke patients (Kim et al., 2021). All these methods involve similar approaches that deal with the clarification or prediction of functional impairment after stroke. Our study finds its place in this context, demonstrating that the presented method can significantly improve the prediction of stroke severity.

Looking ahead, the method has the potential to better support the clinical management of stroke patients by providing a more accurate and individualized approach to predicting outcome and guiding treatment. Notably, the prerequisite for MRI is a FLAIR image, from which the corresponding T1 can be generated (Iglesias et al., 2023). This streamlined imaging protocol could reduce image acquisition time, potentially enabling an earlier start of therapy without any relevant loss of time.

In general, changes in white matter integrity have been correlated with alterations in cortical and subcortical structural connectivity in a range of other neurological conditions. The Network Modification (NeMo) Tool, for instance, has been applied to patient data across a spectrum of diseases, including Alzheimer’s disease, fronto-temporal dementia, normal pressure hydrocephalus, and mild traumatic brain injury, demonstrating the potential applicability of these analytical tools in elucidating and mapping disease-related changes in brain connectivity (Kuceyeski et al., 2013). These precedents underscore the relevance and adaptability of connectivity-based approaches and tools in investigating a wide array of neurological conditions, providing a comprehensive context for understanding the brain’s complex network structure and its implications in disease. The ability of these methodologies to provide nuanced insights into the brain’s structural and functional wiring underlines their potential in advancing our understanding of neurological diseases and refining diagnostic and therapeutic strategies.

It is imperative, however, to acknowledge the limitations of the present work. The use of a normative structural connectome has potential practical limitations. During preprocessing, the creation of manually drawn lesion masks (gold standard) is time-consuming, and the normalization of the masked T1 image and the application of the LST might fail, e.g., due to insufficient lesion segmentation or unsatisfactory lesion filling. It remains to be investigated whether these procedures can be seamlessly integrated into routine clinical practice. Additionally, the objective of our study was not to achieve maximum predictive precision but to explore the feasibility of the probabilistic connectome approach. While we focused on predicting the patient’s current clinical state rather than long-term outcomes, it is worthwhile to discuss the implications of this choice. Predicting the current state is one of the first steps in developing methods to predict future outcomes and can prove valuable in specific scenarios, such as when direct clinical information is unavailable (e.g., due to sedation). However, predicting long-term outcomes is arguably more clinically valuable, requiring an understanding of how acute cerebral disconnections reflect the current state and might forecast future outcomes. Another limitation of our study pertains to the inclusion criteria, focussing on motor-impaired patients, potentially influencing the interpretation of results. By concentrating on this patient group, the precision of lesion size correlation with clinical status might be artificially enhanced compared to a more diverse patient cohort. This specificity in patient selection might also exaggerate the correlation between lesion size and clinical status, as evidenced by discussions regarding the limitations of lesion size as a predictor (Price et al., 2017). It is generally recognised that clinical impairment does not solely stem from the structural lesion but arises from disruptions in the intricate network of brain functions. The conceptualization of brain function as a network operation has propelled multiple studies to utilize network information for outcome prediction. This approach has significantly outperformed models based solely on lesion size and location (Koch et al., 2016, 2021; Kuceyeski et al., 2016). The efficacy of existing acute stroke treatments has also been corroborated through these studies. For instance, systemic thrombolysis has been associated with reduced global network disruption and preserved structural connectivity (Schlemm et al., 2022). It is important to note that our study did not delve into the comprehensive analysis of the entire complex network. This broader exploration, encompassing network measures (Sporns and Zwi, 2004; Bassett and Sporns, 2017; Caliandro et al., 2017) and other techniques like network-based statistics (Zalesky et al., 2010), was not the primary focus of our research. Furthermore, our method was tested on a homogeneous subpopulation comprising single subcortical ischemic stroke lesions. This is in contrast to the great heterogeneity of the overall population of stroke patients to be examined. Consequently, questions arise regarding the extent to which our approach can be extrapolated to cortical lesions and how these can be compared with subcortical lesions. An additional limitation concerns the mirroring of lesion projections in the processing of MRI scans. While this ensures uniform analysis of effects, it disregards the interhemispheric variability inherent in the human brain.

The limitations can be overcome by combining this structural connectome-based approach with longitudinal clinical data. It is also advisable to compare our results with functional connectivity data obtained from stroke patients. Further studies with larger sample sizes should aim to encompass more diverse cohorts, encompassing both cortical and subcortical ischemic strokes. This enhancement of previous knowledge can provide a way for clinicians to apply and intuitively understand additional potential predictors, such as disconnection metrics, in stroke diagnostics and prediction models.

## 5 Conclusion

Our study demonstrates the viability of leveraging probabilistic connectome data to forecast the clinical status of stroke patients effectively. The key advantage of this methodology is its reliance on data that are already routinely gathered in acute stroke care settings. This enables the generation of predictions during the critical phase when initial decisions about reperfusion strategies are being made. As a result, our approach holds the potential to identify patients at a heightened risk for adverse outcomes early on, thereby facilitating timely interventions aimed at enhancing their recovery prospects. Moreover, our method offers the possibility of refining rehabilitation strategies by pinpointing the specific brain regions affected by stroke. Consequently, our findings have important implications for the clinical management of stroke patients.

Further research endeavours should prioritize the validation of our method in larger and more diverse datasets. In addition, exploring the utility of this approach in predicting various dimensions of stroke outcomes, including functional independence and quality of life, is warranted. In future, integrating this information with clinical features and biomarkers in a machine learning approach could lead to the breakthrough of predictive outcome modeling into the realm of clinical decision making.

## Data availability statement

The raw data supporting the conclusions of this article will be made available by the authors, without undue reservation.

## Ethics statement

The studies involving humans were approved by the Ethics Committee, University Hospital Jena, Germany. The studies were conducted in accordance with the local legislation and institutional requirements. Written informed consent for participation was not required from the participants or the participants’ legal guardians/next of kin in accordance with the national legislation and institutional requirements.

## Author contributions

KW: Writing–review and editing, Writing–original draft, Visualization, Validation, Resources, Project administration, Methodology, Investigation, Funding acquisition, Formal analysis, Data curation, Conceptualization. CK: Writing–review and editing, Writing–original draft, Visualization, Validation, Supervision, Software, Resources, Methodology, Investigation, Formal analysis, Data curation, Conceptualization. SB: Writing–review and editing, Supervision, Software, Data curation. FW: Writing–review and editing, Data curation. MS: Writing–review and editing. DG: Writing–review and editing, Data curation. TM: Writing–review and editing, Data curation. FG: Writing–review and editing, Data curation. UT: Writing–review and editing, Data curation. CG: Writing–review and editing, Writing–original draft, Visualization, Validation, Supervision, Software, Resources, Methodology, Investigation, Formal analysis, Data curation, Conceptualization.

## Footnotes

1
https://brain.labsolver.org/hcp_trk_atlas.html
